# Effects of craniectomy on cerebrospinal fluid pressure gradient in the craniospinal space in different body positions of cats

**DOI:** 10.3325/cmj.2026.67.14

**Published:** 2026-02

**Authors:** Marijan Klarica, Gorislav Erceg, Sergej Mihailovič Marasanov, Milan Radoš, Filip Njavro, Darko Orešković, Ivana Jurjević

**Affiliations:** 1 Department of Pharmacology, Zagreb University School of Medicine, Zagreb, Croatia; 2 Croatian Institute for Brain Research, Zagreb University School of Medicine, Zagreb, Croatia; 3 Department of Pharmacology and Croatian Institute for Brain Research, Zagreb University School of Medicine, Zagreb, Croatia; 4 Department of Neurosurgery, Unit for Functional and Stereotactic Neurosurgery and Radiosurgery, Zagreb University Hospital Center, Zagreb, Croatia; 5 Croatian Institute for Brain Research, University of Zagreb School of Medicine and Clinical Department of Diagnostic and Interventional Radiology, Zagreb University Hospital Center, Zagreb, Croatia; 6 Clinical Department of Diagnostic and Interventional Radiology, Zagreb University Hospital Center, Zagreb, Croatia; 7 Department of Molecular Biology, Ruđer Bošković Institute, Zagreb, Croatia; 8 Department of Neurology, Zagreb University Hospital Center, Zagreb University School of Medicine, Zagreb, Croatia

## Abstract

**Aim:**

To assess whether a cranial defect in anesthetized cats alters cerebrospinal fluid (CSF) pressure distribution across the craniospinal axis in different body positions in the setting of intracranial normotension.

**Methods:**

After standardized parietal craniectomy, intracranial and lumbar CSF pressures were simultaneously measured in eight adult anesthetized (α-chloralose, i.p. 100 mg/kg) cats in different body positions: horizontal (0°), head-up (45°; 90°), and head-down (225°; 270°). CSF pressure gradient represents the difference between CSF pressure values in the lateral ventricle (LV) and lumbar subarachnoid space (LSS) in each body position.

**Results:**

At 0°, CSF pressures in the LV and LSS were similar (16.2 ± 0.9 vs 15.8 ± 1.2 cm H_2_O, respectively). In the 90° head-up position, LV pressure was subatmospheric (−1.5 ± 0.5 cm H_2_O), while LSS pressure simultaneously increased (31.4 ± 1.0 cm H_2_O). Conversely, in the head-down 270° position, LSS pressure was negative (−1.7 ± 1.5 cm H_2_O) and LV pressure increased (30.0 ± 1.8 cm H_2_O). Difference between LV and LSS pressures is strongly correlated with hydrostatic difference between the two recording points in the head-up and the head-down position. LV pressure at 90° in the craniectomy group was significantly higher than previously published in cats with an intact cranium in the same position (*P* < 0.001).

**Conclusion:**

Hydrostatic CSF pressure gradient across the craniospinal axis remained unaltered by craniectomy. The finding that craniectomy in the head-up position of animals without intracranial pathology resulted in an intracranial pressure increase may offer additional explanation for the trephined syndrome pathophysiology.

Elevated cerebrospinal fluid (CSF) pressure in the cranium is a particularly dangerous complication of acute brain injury, including traumatic brain injury and malignant cerebral edema (1-4). After physiologic compensatory mechanisms have been exhausted, elevated CSF pressure can impair cerebral perfusion and become life-threatening (1-4). To address this, standardized neurointensive care protocols manage raised intracranial pressure (ICP) in a stepwise fashion using noninvasive or minimally invasive measures aimed at maintaining cerebral perfusion pressure by lowering ICP. These measures include head elevation, optimization of analgesia and sedation, control of body temperature, ventilation strategies aimed at avoiding hypercapnia, hyperosmolar therapy, and, when feasible, CSF diversion procedures such as external ventricular drainage (3,4).

When these measures fail, and ICP cannot be effectively controlled, the condition is termed life-threatening refractory intracranial hypertension, for which last-resort interventions are applied (4). In this scenario, urgent neurosurgical decompressive craniectomy (DC) represents a key therapeutic option; removal of a portion of the calvarial bone converts the closed cranium into a partially “open” space (2,4). DC can rapidly and substantially lower ICP and improve intracranial pressure-volume compensation, thereby “buying time” for other treatments to take effect (2,4,5).

For survivors after DC, once the acute phase of illness has stabilized and ICP has normalized, a new set of problems may emerge. In a non-negligible subset of these patients, new-onset neurological signs and symptoms develop that can no longer be explained by elevated ICP. The clinical scenario instead may be linked to the post-craniectomy calvarial bone defect itself, as an independent pathophysiological factor causing neurological decline in the setting of intracranial normotension. This phenomenon – often described as the syndrome of the trephined or the sunken skin flap syndrome – may remarkably improve after cranioplasty, which suggests a causal role of the bone defect itself and indicates that the integrity of the bony cranium surpasses simple mechanical protection (6-12). A number of mechanisms have been proposed, including direct transmission of atmospheric pressure via the epicranium and dura mater to the intracranial space, and changes in venous outflow and regional cerebral blood flow with consequent alterations in brain metabolism (7-14). However, the complete chain of events in a cause-and-effect manner remains largely unknown (7-10,13,14). More importantly, the basic question remains unanswered: what is the effect attributable to the skull defect itself on craniospinal neurofluids physiology and CSF pressure gradient once ICP has normalized?

To gain additional insight into the impact of cranial bone defects on neurofluids physiology and CSF pressures in the craniospinal space outside the context of intracranial hypertension, we performed a series of experiments on healthy adult anesthetized cats that had undergone a standardized parietal craniectomy. We assessed body position-dependent CSF pressure distribution and compared our findings with previously published data obtained in cats with intact skull anatomy involved in the same experimental protocols (15,16).

## Materials and methods

### Animal experiments

We conducted an experimental study to examine whether a standardized parietal craniectomy impacts the distribution of CSF pressures along the craniospinal axis, as well as craniospinal pressure gradients with respect to body posture. Measurements were obtained in anesthetized cats after parietal craniectomy, across previously defined body positions. To provide a context for correct physiological interpretation, our findings were compared with results from a previously published cohort of cats with intact skulls involved in the same experimental protocols (15-17).

### Ethical considerations

The animals were quarantined for 30 days before the start of the study. All experiments were conducted in accordance with the Croatian Animal Protection Act that was in force at the time the study was performed. The study was approved by the Ethics Committee of the University of Zagreb School of Medicine (04-76/2006-18). The regulations in force at the time allowed for laboratory animals to be obtained from domestic breeders, without written consent. It sufficed for the owners to be orally informed about the experimental protocol. In accordance with good animal research practice, every effort was made to minimize animal suffering, and all invasive and surgical procedures were performed under adequate anesthesia (17).

### Animals and anesthesia

Measurements were performed on adult cats of both sexes, weighing 2.5-5.0 kg. The animals were kept under standardized conditions in cages with natural light–dark cycles and food and water *ad libitum*. To ensure stable measurement conditions, anesthesia was induced with α-chloralose (100 mg/kg, i.p.), as this agent provides long-lasting stable anesthesia with preserved cardiovascular reflexes (17). Invasive arterial pressure was monitored by cannulation of the femoral artery, with occasional blood gas analyses to assess respiratory-metabolic status. Physiological variables remained stable during the measurements.

### Cannulation and CSF pressure recording

To measure cranial and spinal CSF pressures, one cannula was placed in the lateral ventricle (LV) and another in the lumbar subarachnoid space (LSS). After the animal’s head was fixed in a stereotactic head-holder, a stainless-steel ventricular cannula (internal diameter 0.9 mm) was inserted into the lateral ventricle 2 mm lateral and 15 mm rostral to the stereotactic center, 10–12 mm below the dura (17). Lumbar CSF pressure was measured by introducing a plastic cannula into the lumbar compartment: following L3 laminectomy and an incision of the dura and arachnoid, the cannula (internal diameter 0.9 mm) was placed in the lumbar thecal sac. CSF egress was prevented using cyanoacrylate glue as sealant. Dental acrylate was then used to reconstruct the lamina (17).

**Figure 1:**
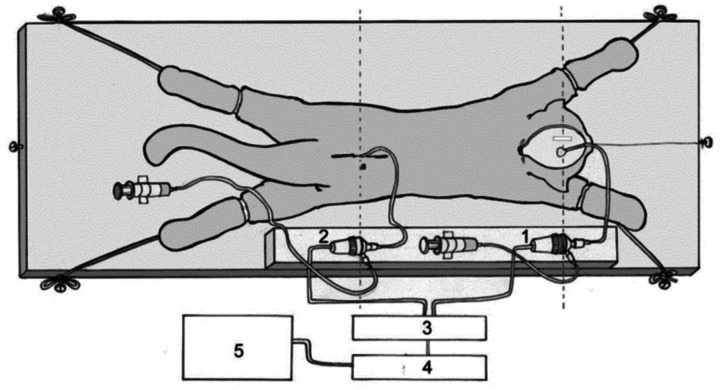
The experimental model. The cat lies prone and secured on a rigid board. The figure shows cannula inserted into the lateral ventricle, the cannula inserted into the lumbar subarachnoid space, and the site of the parietal craniectomy. Dashed lines indicate that pressure transducer membranes are precisely positioned in the same plane as the cannulas inserted into the corresponding cerebrospinal fluid spaces: 1 – pressure transducer connected to the measuring cannula in the lateral ventricle; 2 – pressure transducer connected to the measuring cannula in the lumbar subarachnoid space; 3 – QUAD Bridge; 4-PowerLab/800; 5-computer.

CSF pressure was recorded using standard transducers (Gould P23 ID; Gould Instruments, Cleveland, OH, USA), connected to analog-digital acquisition and computer data storage (Quand Bridge and Power Lab/800, AD Instruments, Castle Hill, NSW, Australia). The transducers were calibrated using a column of water. To ensure adequate measurements in different body positions, the transducer membranes were positioned to match the same hydrostatic height as the tips of the cannulas (Figure 1).

### Experimental parietal craniectomy

Upon placement of the ventricular cannula, a unilateral parietal craniotomy was performed on the contralateral side of the feline skull in an open microsurgical fashion. The cranial defect was performed with a high-speed drill (dimensions approximately 2.0 × 1.0 cm). Care was taken not to violate the underlying dura, prevent CSF leakage, and preserve an intact, pressure-transmissive membrane through which atmospheric pressure could act on the intracranial cavity, analogous to the clinical scenario after DC. The experimental setting is shown in Figure 1.

### Body position protocol

**Figure 2:**
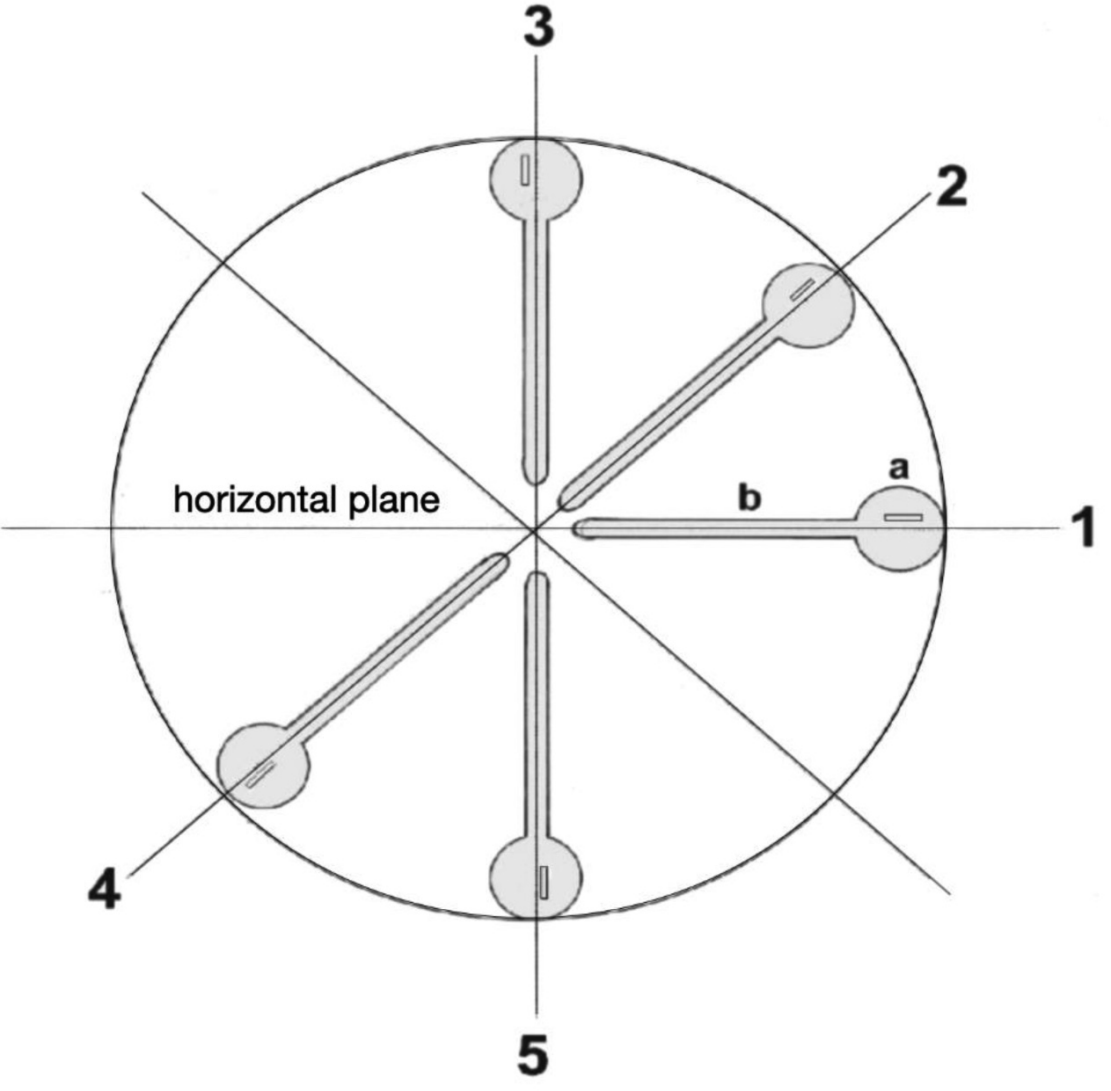
The measurement positions for cerebrospinal fluid pressures in the lateral ventricle and the lumbar subarachnoid space in anesthetized cats with parietal craniectomy. a) cranial compartment; b) spinal compartment; 1 – horizontal (0°); 2 – head up (45°); 3 – head up-(90°); 4 – head down (225°); 5 – head down (270°).

Following cannulation, positioning in the stereotactic apparatus, and craniectomy, each animal was secured on a rigid board. Details of the restraint procedure have been described previously (16). The board with the animal was then sequentially positioned in five standard positions: 0° (horizontal), 45° head-up, 90° head-up, 225° head-down, and 270° head-down (Figure 2).

Each position was held for sufficient time to allow the stabilization of CSF pressure as previously described (16), and both intraventricular and lumbar CSF pressure values were recorded. Ventricular pressure values in the 90° upright position were compared between our series and a previously published series of cats with an intact cranium (17).

### Statistical analysis

Data are shown as mean ± standard error of the mean. The effect of body position on pressures was assessed using a repeated-measures method, with correction for multiple comparisons when applicable. Ventricular pressures at 90° were compared using an unpaired-samples *t* test. *P* < 0.05 was considered statistically significant. All statistical analyses were performed using SPSS Statistics for Windows, version 20.0 (IBM Corp., Armonk, NY, USA).

## Results

### CSF pressure in cats with craniectomy in different body positions

**Figure 3:**
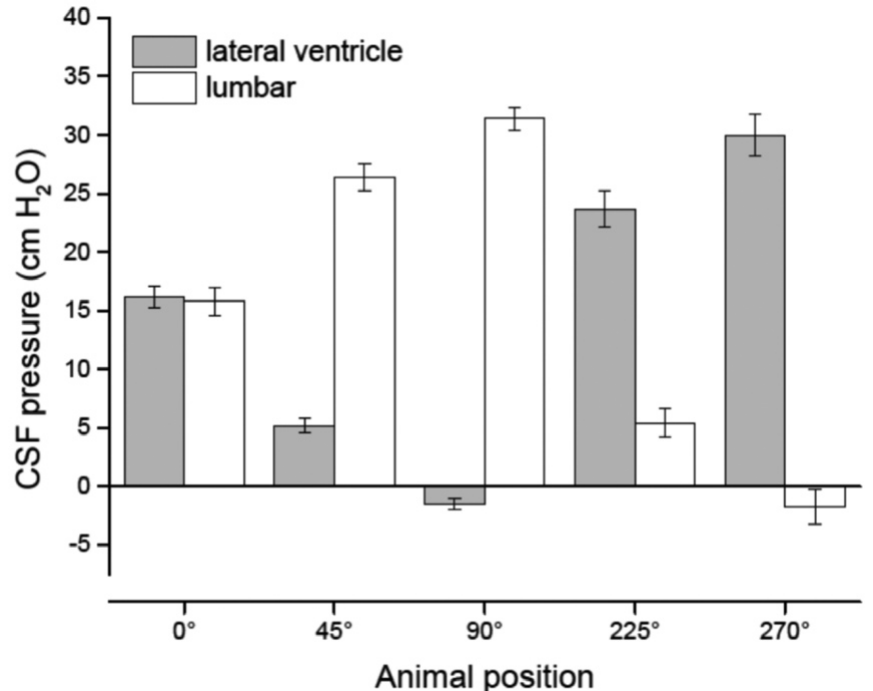
Cerebrospinal fluid (CSF) pressure values in the lateral ventricle (gray) and lumbar subarachnoid space at the L3 level (white) measured in various positions (horizontal 0°; head up 45°; head up 90°; head down 225°; head down 270°) of anesthetized cats with a parietal craniectomy of approximately 2 × 1 cm in size, secured on a wooden board. The x-axis shows the positional angles (°) representing the position of the animal in relation to the horizontal plane. The y-axis shows CSF pressure values in centimeters H_2_O. The bars represent the mean values of the measurements, and the vertical lines are the standard errors.

In the group of eight cats subjected to a standardized parietal craniectomy, changes in body position led to marked reciprocal changes in CSF pressures between the cranial and lumbar compartments. LV and LSS CSF pressures in all measured positions are shown in Figure 3. In the horizontal position (0°), LV and LSS pressures were similar (16.2 ± 0.9 vs 15.8 ± 1.2 cm H_2_O, respectively). In the head-up 45° position, LV pressure decreased (5.2 ± 0.6 cm H_2_O), while LSS pressure increased (26.4 ± 1.2 cm H_2_O). In the head-up 90° position, LV pressure became negative (−1.5 ± 0.5 cm H_2_O), while LSS pressure increased further (31.4 ± 1.0 cm H_2_O). Conversely, in the head-down 225° position, LV pressure increased and LSS pressure decreased (23.7 ± 1.6 cm H_2_O vs 5.4 ± 1.2 cm H_2_O). In the head-down 270° position, LSS pressure reached negative values (−1.7 ± 1.5 cm H_2_O), while LV pressure further increased (30.0 ± 1.8 cm H_2_O) (Figure 3).

### Comparison to animals with intact cranium in the head-up position

In a previous experiment in cats with intact skulls (17), in which LV and LSS CSF pressures were measured using the same protocol, LV pressure in the 90° fully upright head-up position was subatmospheric (−3.9 ± 0.3 cm H_2_O) (17). In our series, LV pressures at 90° were also negative, but closer to atmospheric values (−1.5 ± 0.5 cm H_2_O; Figure 3). If we directly compare the two cohorts, cats with craniectomy (n = 8) had significantly higher ventricular CSF pressure than cats with an intact cranium (n = 4) (*P* < 0.001).

## Discussion

We are normally spending two thirds of our life-time in the head-up position. The same is valid for patients with DC after the acute phase of illness has stabilized. Our results suggest that intracranial CSF pressure of animals with craniectomy in the head-up position is higher than in normal subjects without a cranial defect. Thus, it seems that ICP in patients with DC after acute phase of illness is higher than in healthy subjects.

After a change of body position from supine to upright, a CSF pressure gradient forms inside the craniospinal space with normally very low (often negative) values in the cranium and highly positive values in the LSS (15-19). Our results suggest that CSF pressure gradient is still present inside the craniospinal space after craniectomy but with a significant change of CSF pressure values in two recording points.

### Effect of changes in body position on CSF pressure

Frequent changes in body position are a defining feature of daily human life. Since these shifts are accompanied by posture-dependent CSF pressure gradients, these gradients have been explored in clinical and experimental settings for many decades (20-22). Early classical works described the influence of body posture (eg, recumbent vs sitting) on lumbar CSF pressure and introduced concepts such as the hydrostatically indifferent point, providing a framework for understanding postural pressure changes along the craniospinal axis (18,19). It has also long been recognized that head elevation in neurosurgical patients can affect cerebral perfusion not only through hemodynamic factors, but also partly via posture-dependent changes in ICP (3). Namely, elevating the head relative to the body leads to a decreased intracranial CSF pressure. For this reason, in clinical practice, patients with intracranial hypertension are often positioned with the head elevated (mostly by 30°).

The classical concept of CSF physiology emphasized active production and passive absorption of CSF as the primary determinants of CSF pressure, assuming relatively constant and stable relationships within the closed craniospinal system (15-17). In contrast, many recent clinical and experimental studies have shown that changes in body position predictably change CSF pressure distribution. For example, in the upright position intracranial CSF pressure can become subatmospheric, and this change may represent a sustained physiological state rather than a transient phenomenon (18-21). Such findings challenge simplified classical interpretations of a strictly unidirectional secretion-circulation-absorption scheme and have supported alternative concepts emphasizing distributed hydrodynamics and pressure-volume interactions within the craniospinal system (23-26).

Experiments in cats with intact cranial integrity and uncompromised craniospinal communication provide a representative example of how body position influences CSF pressures. In these experiments, a reciprocal response is observed between the cranial and spinal compartments. In the head-up position, ventricular CSF pressure falls to subatmospheric levels, while lumbar pressure rises; in the head-down position, the situation is exactly opposite (15,16,27). All this suggests that CSF pressure along the craniospinal axis behaves as a hydrostatic pressure, as changes in pressure values at the measurement sites preserve the hydrostatic difference. In addition, this observation challenges the classical concept of CSF circulation, since it raises the question of how CSF could circulate from the cranium into the spinal subarachnoid space, opposite to such a large hydrostatic gradient with the lowest pressure in the cranium and highest in the LSS.

### Changes in CSF pressure after craniectomy

An increased CSF pressure in the LV of adult cats with craniectomy in the upright position could, as is generally stated, be attributed to the influence of atmospheric pressure on the intracranial space. However, such an effect could only occur if the atmospheric pressure exceeds the values of intracranial pressure. Following DC, the bony skull defect introduces a specific environment in which the intracranial compartment is no longer completely isolated from the direct impact of atmospheric pressure (7-14). By acting through the soft-tissue membrane over the defect, atmospheric pressure can influence intracranial mechanics, particularly when hydrostatic relations during verticalization promote the development of subatmospheric intracranial pressure (7-14). In the head-up scenario, it is reasonable to suppose that negative intracranial pressure is dampened toward atmospheric values, as the pressure difference between the intracranial space and the atmosphere is partially compensated through the compliant soft-tissue interface (7-14).

Our data support this line of reasoning: while in the head-up position ICP remained negative after craniectomy, it shifted toward atmospheric values compared with cats with intact skulls (17). However, experiments involving micro-volume changes of spinal CSF volume in cats indicate another possible mechanism causing higher intracranial CSF pressure during the upright position of animals with a cranial defect (17). Specifically, LV pressure increases when the spinal CSF volume is increased and decreases when the volume is reduced. Spinal CSF volume during upright positioning of animals with a craniectomy could increase due to a shift of CSF from the cranial to the spinal compartment under the influence of gravity and the atmospheric pressure. The spinal compartment would then be more filled, which could exert a cranial shift of the hydrostatically indifferent point and point of zero pressure (18,19).

In our study, the postural relationship between ventricular and lumbar pressures remained unchanged (15-17). This implies that the skull defect modulates the absolute level of ICP, while the mechanism generating posture-dependent craniospinal pressure gradients remains qualitatively preserved (15,16,18,19,22). This interpretation has clinical relevance, as it suggests that even after acute intracranial hypertension is normalized, the defect itself can modify intracranial physiology during daily postural changes. In this way, it may contribute to the pathophysiology of the trephined syndrome, which develops in the setting of intracranial normotension and may improve after cranioplasty (6,11,12).

Although our data do not allow for direct conclusions on the effect of craniectomy on cerebral perfusion, posture-dependent changes in ICP with influence on the pressure gradient between blood capillaries, interstitial fluid, and CSF, represent a plausible link that could influence cerebral perfusion and brain metabolism (3,28). In this context, cranioplasty not only restores skull rigidity and provides mechanical protection to the brain, but also influences intracranial physiology, including cerebral perfusion and ICP-related parameters, thereby providing a basis for neurological recovery (28-32).

This study has several limitations. First, the data for the control group of cats with intact skulls were taken from a previously published cohort and are therefore inferior to data that could be obtained in the same animals before and after craniectomy at different body positions. Second, we did not perform direct imaging or volumetric analysis of CSF volume changes in different compartments or simultaneous measurements of cerebral blood flow, which makes our conclusions indirect. Further research measuring these parameters in the same subjects, including volumetric and hemodynamic data, is warranted to directly validate the proposed mechanisms.

In conclusion, our results suggest that a standardized parietal craniectomy does not interfere with posture-dependent craniospinal CSF pressure gradients, but rather changes the absolute level of intracranial pressure. Although the reciprocal postural pattern of ventricular and lumbar pressures remained almost unaffected, the ventricular pressure in the upright position was closer to atmospheric pressure compared with values in the intact skull. These results further support the new concept of CSF hydrodynamics and provide a physiological framework relevant for understanding the trephined syndrome, in which symptoms develop after a resolution of intracranial hypertension and may improve after cranioplasty.
